# Recruitment of Community College Students Into a Web-Assisted Tobacco Intervention Study

**DOI:** 10.2196/resprot.6485

**Published:** 2017-05-08

**Authors:** Scott McIntosh, Tye Johnson, Andrew F Wall, Alexander V Prokhorov, Karen Sue Calabro, Duncan Ververs, Vanessa Assibey-Mensah, Deborah J Ossip

**Affiliations:** ^1^ Department of Public Health Sciences University of Rochester School of Medicine & Dentistry Rochester, NY United States; ^2^ School of Education University of Redlands Redlands, CA United States; ^3^ MD Anderson Cancer Center Department of Behavioral Science The University of Texas Houston, TX United States

**Keywords:** community colleges, tobacco control, study recruitment, qualitative research, baseline

## Abstract

**Background:**

United States college students, particularly those attending community colleges, have higher smoking rates than the national average. Recruitment of such smokers into research studies has not been studied in depth, despite a moderate amount information on study recruitment success with smokers from traditional four-year colleges. Recruitment channels and success are evolving as technology evolves, so it is important to understand how to best target, implement, and evaluate recruitment strategies.

**Objective:**

The aim of this paper is to both qualitatively and quantitatively explore recruitment channels (eg, mass email, in-person referral, posted materials) and their success with enrollment into a Web-Assisted Tobacco Intervention study in this priority population of underserved and understudied smokers.

**Methods:**

Qualitative research methods included key informant interviews (n=18) and four focus groups (n=37). Quantitative research methods included observed online responsiveness to any channel (n=10,914), responses from those completing online screening and study consent (n=2696), and responses to a baseline questionnaire from the fully enrolled study participants (n=1452).

**Results:**

Qualitative results prior to recruitment provided insights regarding the selection of a variety of recruitment channels proposed to be successful, and provided context for the unique attributes of the study sample. Quantitative analysis of self-reported channels used to engage with students, and to enroll participants into the study, revealed the relative utilization of channels at several recruitment points. The use of mass emails to the student body was reported by the final sample as the most influential channel, accounting for 60.54% (879/1452) of the total enrolled sample.

**Conclusions:**

Relative channel efficiency was analyzed across a wide variety of channels. One primary channel (mass emails) and a small number of secondary channels (including college websites and learning management systems) accounted for most of the recruitment success.

**Trial Registration:**

ClinicalTrials.gov NCT01692730; https://clinicaltrials.gov/ct2/show/NCT01692730 (Archived by WebCite at http://www.webcitation.org/6qEcFQN9Q)

## Introduction

Community colleges are a unique setting with an understudied population consisting of distinct subpopulations, including young adults, older nontraditional students, armed services veterans, individuals with full time jobs, and single parents. Rates of nonwhite students and students with low socioeconomic status (SES) are higher in community colleges compared to traditional colleges and universities [1-3]. Of all college students, nearly half (42-45%) attend community colleges [4,5]. The total number of enrollees at community colleges in the United States was approximately seven million during the 2013-2014 academic year, and according to a recent report, it is estimated that enrollment at community colleges is projected to increase by 21 percent to 8.2 million students between 2014 and 2025 [6].

Community colleges have increasingly become more popular for many groups: young adults, as a pathway to costlier traditional colleges and universities; veterans, as an increasingly available option upon returning from active duty; and nontraditional older adults seeking to improve their job market qualifications [7]. President Barack Obama launched the American Graduation Initiative in 2009 and America’s College Promise in 2015 to increase enrollment and provide new avenues for persons to successfully enter the work force [8,9].

Cigarette smoking prevalence is higher among community college students (as high as 34%), compared to their traditional college and university counterparts [10-12]. Although the community college population is projected to grow, research studies that target this diverse student body remain limited [13-16]. Community college students are more vulnerable to health risks compared to other college students [3,17], yet limited research has been undertaken on smoking cessation interventions in this population [13,18]. Most of the relevant research on smoking cessation has focused on traditional colleges and universities, or those institutions in combination with community college populations [14].

The limited studies that are available indicate that community college students (compared to other college students) are more likely to smoke, describe themselves as regular smokers, and be unsuccessful at quitting [14,19]. Community college students report being less concerned about smoking-related health problems than other college students [11], and nearly half (45%) state that quitting smoking would have little or no impact on their health [20]. Such findings strongly support the need for research in this vulnerable population to explore effective smoking cessation interventions.

Challenges exist when applying research from traditional college and university environments to the environments of community colleges, which can be underscored by distinct characteristics of the community college mission, organizational capacity, and student population [21]. Although published literature reports demographic differences between students at community colleges and traditional colleges and universities, little is known about the nature of successful recruitment strategies to engage such students into randomized controlled trials (tobacco cessation intervention or otherwise).

An updated review of Web-assisted tobacco interventions in The Cochrane Database of Systematic Reviews included a total of 28 studies with over 45,000 participants for interventions that varied in intensity and outreach [22]. Studies were included with information on recruitment of various populations, including adults from the general population, adolescents, and university students. In most studies, recruitment was Web-based (search engines and browsing), while several trials also used press releases, billboards, television advertisements, posters, and flyers, in addition to Web-based strategies. Using these recruitment methods, participants included in these trials were smokers who were motivated to quit and chose the Internet as a tool for smoking cessation support.

Recruitment channels that have demonstrated particular success vary greatly by study design and population, and data on direct comparisons of channel performance are not often reported. As reported in the 2013 Cochrane review [22], online recruitment accounts for most participants in online cessation studies, whereby participants find the studies through search engines and browsing. These studies reported a wide variety of other recruitment channels, including: traditional materials (flyers, posters, brochures, school newspaper ads and articles, school-wide announcements, newspapers, health plan magazine advertisements, press releases, billboards, bus interior posters), engagement with champions (classroom presentations, school liaison referrals, employee mailings, physician referrals), face-to-face (lunch-hour sign-up tables, word of mouth, medical clinics), and electronic media (website banners and links, purchased email addresses, electronic newsletters, social network sites and campaigns, online smoking cessation forums, television, radio, online research panels, search engines, central telephone numbers, and mass emailing).

The present study explored research study recruitment strategies in two phases of a randomized controlled trial (ClinicalTrials.gov NCT01692730). The initial qualitative phase (Phase 1) provided key formative evaluation information to aid in the design of the study’s specific recruitment methods. The quantitative phase (Phase 2) assessed the study’s success with these methods from initial reach to ultimate enrollment via recruitment channels by examining (1) initial recruitment of potential participants, (2) those who were recruited and consented, and (3) those who consented and were ultimately enrolled and randomized.

## Methods

Proposed research study recruitment channels (traditional channels such as flyers and posters; digital channels such as mass emails, digital bulletin boards, and websites) were initially examined qualitatively with community college students and staff. The Phase 1 study protocol, conducted as planned formative research, was approved by the principal investigator’s institutional review board (IRB), and informed consent was obtained from all participants. Phase 1 qualitative monetary incentives included US $15 for key informant interviews (KIIs), and US $25 for focus group (FG) participation, while Phase 2 randomized trial monetary incentives included no payment for baseline enrollment (in order to minimize recruitment of individuals who might falsify smoking status and/or cessation intent in order to receive a payment), and increasing incentivization for follow-up outcome questionnaires (in order to maximize retention of valid participants) at 1-month (US $10), 6-month (US $15), and 12-month (US $20) timepoints.

### Phase 1: Qualitative Methods

Research sites included four Western New York community colleges, selected through purposive sampling to include two in rural areas, one in a middle-class suburb, and one in a central urban area. Qualitative research methods based on our previous research protocols, and consistent with evidence-based strategies [23], included KIIs (n=18) and four FGs (n=37) with a total of 55 students, administrators, and staff. Potential respondents were reached via emails from campus champions, courseware postings, and hard-copy flyers with IRB-approved wording. All respondents who presented at prearranged times were provided with information sheets describing the goals of the research, and how the Phase 1 qualitative work would inform key elements of the Phase 2 randomized trial. Each participant provided informed, signed consent.

Purposive sampling was utilized for the KIIs, which were conducted with students (n=11; smokers and ex-smokers, traditional, nontraditional, and veteran students), and with faculty and staff (n=7). Two male PhD level senior researchers (SM, AW), each with more than 10 years of experience conducting qualitative research methods, participated in all KIIs: one acted as a facilitator while the other was a cofacilitator/note taker. Semistructured KII interview guides were developed, pretested, and pilot tested (as were guides for FGs), with domains including smoking, smoking cessation, campus tobacco-free policies, and the study’s specific proposed research recruitment channels. Qualitative data collection progressed iteratively, with early interview responses used to refine subsequent KII and FG interview guides, and protocols for interviews and FGs. Open-ended questions in the guides were designed to elicit qualitative responses related to the targeted domains. Each KII and FG was audio recorded, and researchers made field notes before and after all contact with participants. Most of the students interviewed were daily commuters living off-campus. Seven KIIs were conducted with nonstudent campus opinion leaders, including student health center directors, administrators, educators, an associate dean, and a director of residential life. Faculty and staff were interviewed in seven KII sessions (n=9; two of these were conducted with two interviewees in attendance), which consisted of two health center directors, one residential life director, four student service administrators, one faculty from the department of nursing, and one associate dean. Only researchers and respondents were present at all KIIs (lasting between 30 and 60 minutes each) and FGs (lasting 90 minutes each).

The four FGs, assembled with purposive sampling as described above and whose members were similarly provided with information sheets and informed consent procedures, were conducted with 8-12 students each, inclusive of students who were smokers or ex-smokers varying in race, age, gender, and student type (traditional young adults, nontraditional older adults, and veterans). The FGs addressed similar topics as those in the KIIs. Although all KII participants were invited to the FGs, per our previous protocols and for the purposes of efficient outreach and iteratively building on qualitative findings, only five of the FG participants had previously participated in KIIs. Aside from these five individuals, no other repeat interviews were carried out. One FG consisted of students who lived on-campus.

The KIIs and FGs used iteratively refined interview guides with open-ended questions addressing the domains of tobacco use, campus polices, and cessation resources that are described elsewhere [24], as well as domains addressing recruitment strategies, materials, and channels, which are examined in the present analysis. See Multimedia Appendix 1,Multimedia Appendix 2,Multimedia Appendix 3, and Multimedia Appendix 4 for examples of the key recruitment materials that were refined during this process and later used in the Phase 2 randomized trial. See Multimedia Appendix 5 for an example of a KII interview guide and Multimedia Appendix 6 for an example of an FG guide.

#### Materials Presented in Key Informant Interviews and Focus Groups

Materials were presented during KIIs and FGs to elicit reactions, opinions, and recommendations for recruitment success (ie, success in recruiting and enrolling participants into an online research study) with a population of community college students. Examples of recruitment channels were identified from the limited literature available for this population, and from the study team’s previous research recruitment strategies [18,25-30]. Multimedia presentation of materials included hard-copy mock-ups of passive recruitment materials (flyers, tent-cards), and overhead displays of pictures of various recruitment channels, including electronic methods (eg, Quick Response [QR] codes, email invitations, electronic bulletin board displays). Samples of visual materials included variations of persons’ faces, *no smoking* signs, and other iconic images, to elicit feedback and discussion. These proposed mock-ups, displays, and associated procedures were considered initial drafts, and participants were informed that their feedback was a vital component of the overall research by helping to make the study methods maximally relevant to the intended participants.

#### Analysis of Qualitative Data

The Grounded Theory Approach guided the present analysis, specifically employing a Constant Comparative Method, in which data collection and analysis progressed iteratively [31-34]. The content for the KII and FG guides began with open-ended supposition-building questions concerning research participant recruitment (specifically, community college student interest in, and likely methods for, successful recruitment into a randomized trial). After each KII, questions were refined based on new suppositions informed by the analysis of the rich content provided by respondents. As the analysis progressed, iteratively gathered data supported the ongoing theoretical sampling of general subgroups of participants (young adults, midlife and older students, and military veterans). Data saturation that supported emerging themes was observed to have been reached in both the KII and FG processes.

Five project team members conducted open coding and then axial coding of each recorded interview. After establishing a broad framework for data analysis (open coding of themes and specific quotes related to recruitment channels and to features of online smoking cessation interventions), axial coding (a structured process to associate self-reported constructs) led to the development of specific categories and subcategories [31]. Following code creation, quotations of text with codes were placed in a spreadsheet to aid in analyses. Project staff debriefed and compared emerging data patterns to solidify codes, categories, and subcategories, and resolved discrepancies. Two coders then independently coded quotations of text per identified theme categories (eg, perceptions of potential effectiveness of a given recruitment channel), and Cohen κ was run to quantify the agreement between these two independent raters.

### Phase 2: Randomized Trial

#### Participants in Web-Assisted Tobacco Intervention Trial

To be eligible for the Web-Assisted Tobacco Intervention (WATI), participants had to be enrolled in a community college, smoke at least 5 cigarettes per week, and desire to quit smoking within the next 3 months. All potential participants reached an online survey with a description of the study, followed by an online consent process, enrollment instructions, the study’s baseline questionnaire, and access to the appropriate study intervention website (see below).

The Phase 2 study protocol was approved by the principal investigator’s IRB, and informed consent was obtained from all participants. The parent IRB-approved process, and the nature of the study design, did not require each participating campus to complete their own site-level IRB approval process (it was not considered a partnering research initiative, as recruitment could occur outside of campus environments). However, some community colleges (individually, or via larger organizational review in systems with multiple community colleges) deemed this necessary, resulting in successful IRB approval (or exemption) obtained from eight community college systems, representing over 30 campuses. IRB requirements included a combination of (1) the provision of a copy of the parent IRB approval letter for review, (2) submission of a short application, and (3) the provision of all materials previously reviewed and approved by the parent IRB.

#### Baseline Questionnaire

A baseline questionnaire was developed from our previous smoking cessation studies that used technological interventions, understudied populations, and multiple recruitment channels [21,27,29]. The questionnaire was pretested with multiple versions that were improved iteratively with feedback from: (1) a group of students from a local community college (a health class of approximately 40 students); and (2) for comparison purposes and ongoing feedback from young adult college age students, a convenience sample of three local university students working as independent study students on the parent study. Pretesting instructions elicited feedback with respect to length of time, understandability, and recommendations for changes.

#### Recruitment

All online data were captured using Research Electronic Data Capture (REDCap), a widely-used data capture application developed for large-scale research projects [35]. Those who qualified and consented proceeded to the baseline questionnaire, after which they were randomized and enrolled in one of two intervention arms of the parent WATI study, as described elsewhere [13]. Interventions varied in level of interactivity, but both included Public Health Services Guideline-based tobacco cessation information [36], and information and formatting used successfully in other studies [37].

Process evaluation strategies for the recruitment phase of the parent study primarily involved monitoring dates of implementation of various channels (eg, the date a community college would send a mass email to their student body, or a campus website began recruitment advertising for the study), and rates of accrual to study enrollment. Based on the slower than expected rate of recruitment from an initial sample of 16 community colleges in Western New York, the target sample of colleges was expanded to recruit participants from any community college in the state of New York, and eventually from any US state (except the 27 states whose tobacco cessation websites were developed by the same vendor in the parent study).

Channel efficiency was defined as the percentage of those reached who ultimately enrolled for each channel. Individual channel reach and efficiency are reported, including all channel exposures as self-reported by participants and including only the single most influential channel per self-report.

#### Recruitment Categories and Channels

##### Traditional Materials

Building on successful strategies in our previous research [27,29,30], materials and strategies tested in Phase 1 of the present study included flyers with tear-offs, posters, table-tent cards (for cafeteria tables and tabling events), business-sized cards (for tabling events, college orientation, and college health centers), and variations on these *passive recruitment* hard-copy materials which were easy to produce and ship to campus champions.

##### Engagement With Community College Champions

To determine which materials, electronic strategies, or other student-engagement strategies were available and accessible, at least one campus champion from each study location was engaged and contacted regularly. Typically, champions were in the offices of the President, Student/Academic Affairs, Student Activities, or Student Health. It was determined that despite previous commitments of support for the present study from the institution (eg, letters of support at the time of funding applications), there was little if any *institutional memory* of the nature of the study, which resulted in newly beginning the engagement process with most campuses. Engaged champions assisted with mass emails, placement of recruitment ads in electronic media, coordination of hard-copy recruitment materials, and assistance with site-specific requirements (materials approvals, IRB approvals, and administrative approvals). Ongoing engagement consisted of phone calls, emails, and regular newsletters with information on recruitment success and study goals. Support from officials at higher levels was also solicited, such as those from multicampus networks (eg, State University of New York, New York Community College Trustees, Illinois Council of Community College Administrators, Kentucky Community and Technical College System).

##### Face-to-Face

Investigators periodically engaged in face-to-face events such as student meetings, classes, and tabling events (eg, health fairs) on campus. Given that only 16 campuses were readily accessible by the research team in the study’s immediate geographic region, these more time- and resource-intensive activities were primarily limited to nearby campuses.

##### Electronic Media

This category includes at least two instances of radio Public Service Announcement advertisements and fewer than 10 earned media events on local or statewide radio and television (eg, interviews regarding the study, or related tobacco control stories with opportunities to broadcast the study’s contact information). However, this broad category primarily consists of electronic bulletin boards, computer screens on campus (eg, wallpaper and computer time-out messages), mass emails (eg, *email blasts*, *global emails*) sent to a college’s entire student body, and placement of a recruitment ad on a college website or specific *courseware*, also known as *learning management systems* (LMSs) such as BlackBoard or Genesis.

#### Enhanced Recruitment Strategies

In addition to the more traditional recruitment strategies described above, additional strategies were employed to enhance specific channel success (eg, adding multiple contact options, expanded use of digital strategies) and to provide the project with additional data sources (specifically, multiple REDCap datasets) for both recruitment and retention in the WATI trial. Additionally, a specific aim of the WATI study was to investigate successful recruitment strategies that could inform future research (not just smoking cessation trials). Therefore, protocols and channels were implemented to provide multiple opportunities for capitalizing on digitally-provided contact information.

##### Posters With Quick Response Codes

Posters and flyers with QR codes were made available to community college campuses. Potential research subjects could scan the QR codes with a smartphone to access the study. Students using this channel entered their contact information into a specific REDCap database, and were offered a link to the baseline and consent processes to input their email address into an online registry (another REDCap database; see below). The research team accessed these registries to send additional direct emails with a link to the baseline enrollment survey.

##### Online Registry

Potential participants who were not eligible at the time of initial online screening were offered the option of consenting to enroll in a wait-list registry, for the purposes of being contacted for the present study as well as future studies. To serve both the present trial and future trials, this protocol was separately approved by the principal investigator’s IRB, and separate online informed consent was obtained from all participants. Regular monitoring of the data in this registry allowed project staff to identify persons who, depending on the reasons they were deemed ineligible at the time of initial screening, were likely to now be eligible. These instances include individuals who were 17 years of age at the time, or who were not ready to quit smoking within a three-month time frame. Such registry participants were recontacted periodically and offered new screening for the WATI trial. Other reasons for student ineligibility into the WATI study, but valid for potential inclusion in this broader registry for potential research participants, were that individuals were not currently smokers, smoked too infrequently, or were not community college students.

##### Enrollment Completion Reminders

To complete enrollment, eligible individuals had to register with the intervention website and then log into the website at least one time within 72 hours of registration. These two vendor requirements became barriers to enrollment. The study’s research team used phone calls and emails to remind potential enrollees to complete their enrollment by registering and logging in.

##### Contacting Community Colleges

Contacts were attempted with numerous administrators at numerous community colleges around the country. A targeted strategy was used to maximize efficiency. First, an online search was conducted, focusing on community colleges located in states with a high prevalence of smoking and those with tobacco and/or smoke-free policies. Once a list of community colleges was generated, relevant contact information was obtained for the appropriate administrators (eg, Dean of Students, Vice President of Student Affairs, Administrator on the Smoking Policy Committee). When contacted, the details of the study were discussed and these contacts were followed up with appropriately to provide recruitment materials and to assist with any necessary IRB approvals.

##### Re-engaging Noncompleters

A participant was fully engaged as a research participant after completing all enrollment tasks and logging into the intervention website for the first time. Dropouts were defined as persons who stopped engagement with the enrollment process prior to this initial log-in. Such persons dropped out (1) during the online consent and baseline process, or (2) at some point during the website vendor’s multi-step process. Such participants were identified as noncompleters, and were recontacted by email and phone and, depending on when the drop out occurred (did not complete baseline, did not initially register with vendor, did not receive or retrieve email from vendor with log-in instructions, did not complete log-in with correct log-in credentials, or did not have correct log-in credentials), project staff would re-engage the participant to again initiate enrollment.

Each of the above strategies was considered a flexible and adaptive component of an overall proactive recruitment strategy. Changes were considered responsive to formative evaluation results (Phase 1 findings), as well as process evaluation findings (relative success in the context of expected results, such as how well a recruitment channel is working). This adaptableness was purposefully built into the 5-year study design to accommodate the need to improve or refine recruitment strategies based on observed barriers and recruitment rates. Specifically, an initial small number of recruitment sites (four community colleges) allowed the project team to more intensively assess effective strategies and refine procedures. Early feedback from these four campuses that yielded poor recruitment led to a relaxation of participant inclusion criteria: from 10 cigarettes per day in the first few months of the study, to the inclusion of intermittent smokers. By carefully monitoring recruitment rates, two other key changes to methods were made to reach recruitment goals: (1) establishing a longer recruitment timeline (a full year was needed to reach the target sample size), and (2) expanding the targeted geographic regions to ensure adequate reach within the target population. The study expanded from 16 targeted community colleges to all community colleges in the State of New York, and then to all community colleges in the United States where the state quitline and quitsite were not managed by the study’s intervention vendor (resulting in 27 eligible states).

## Results

### Phase 1: Qualitative Research

Cohen κ was run to determine if there was agreement between the two independent raters on whether 138 individual comments from FGs and KIIs were identified as *electronic recruitment* channels (eg, mass emails, website banner ads, LMS/courseware, QR codes, electronic bulletin boards), *passive recruitment* channels (eg, flyers, posters, table-top displays); or *personal recruitment* channels (eg, word of mouth, referral to the study). There was strong agreement between the two raters' judgments across all three categories: electronic recruitment, κ=.734 (95% CI 0.616-0.852,  *P*<.001); passive recruitment, κ=.944 (95% CI 0.882-1.000,  *P*<.001); and personal recruitment, κ=.708 (95% CI 0.532-0.885,  *P*<.001).

No response differences or inconsistences were observed by interviewers or coders based on campus location (rural, urban, and suburban). As can be seen in Multimedia Appendix 7, illustrative quotes capture the nature of perceptions in each of the three overall domains (electronic, passive, and personal), and some key specific recruitment channels, particularly the use of global (mass or blanket) emails to students, the use of LMS, websites, and the more traditional channels of flyers, tabling, and personal referrals. Interestingly, some administrators’ skepticism in students’ regular use of campus mail (which contrasts with students’ endorsements of this channel) helps to provide context for subsequent administrative barriers to allow for mass emails, vis-à-vis the relative success in recruitment of students (see below).

### Phase 2: Randomized Trial

#### Channel Reach

Placement of hard-copy materials (eg, flyers, posters, table-top tent cards) ranged greatly between campuses, from as few as one initiative with only one strategy at the beginning of one semester to high levels of engaged placement every semester across four consecutive semesters or more. Similarly, digital strategies ranged from as few as one website placement or one mass email to high levels of engaged recruitment efforts, including sustained placement of website and LMSs and mass emails multiple times per semester across four consecutive semesters or more. Precise numbers of exposures were only available from those campuses with supportive champions, while overall exposures to channels were primarily assessed by self-report from campus contacts and observable increases in recruitment activity, as measured by the various REDCap databases, consents, and enrollments.

Students responded from a total of 82 community colleges; 38 from the State of New York, and 44 from additional states when recruitment was expanded nation-wide, including Alabama, Arizona, California, Illinois, Indiana, Kentucky, Maine, Michigan, Minnesota, Nevada, New Jersey, Pennsylvania, South Carolina, Tennessee, Washington, and West Virginia. Across all study recruitment channels, a total of 10,914 potential participants were initially reached. This initial *reach* is defined by the total number of independent first-time communications with the study (emails and phone calls to the study coordinator, or completion of the online screener). These communications were facilitated by the study's recruitment channels, which directed subjects to one or more of these contact methods (email address, phone number, link to online screener). Of these, 2696 individuals (2696/10,914, 24.70%) completed the online consent process, and 1452 (1452/10,914, 13.30% of those reached; and 1452/2696, 53.86% of those consented) were successfully enrolled to the WATI study. Measures of the success of the recruitment channels were defined as percentage of those reached who qualified and consented to the study (2696/10914, 24.70%) and, from these, the percentage who completed all steps for full enrollment (1452/2696, 53.86%).

As described earlier, channel efficiency was defined as the percentage of those reached who ultimately enrolled for each channel. Individual channel reach and efficiency are reported including all channel exposures, as self-reported by participants (Table 1) and including only the single most influential channel per self-report (Table 2). Specifically, participants were asked two survey questions, each with drop-down choices of all recruitment channels. The first question, in which participants could select *all that apply* read, “How did you find out about this research study?” The second question read, “Of these, which one was most effective in getting YOU to join?”

**Table 1:** Channel reach (multiple channels reported).

Channel	Initially Reached (n=10,914)	Completed Consent (n=2696)	Enrolled and Randomized (n=1452)	% Reached Who Then Consented	Channel Efficiency (%)	% of Total Enrollees
Email	6139	1927	973	31.39	15.85	67.01
Courseware message (Blackboard, Angel, Genesis, etc)	769	309	176	40.18	22.89	12.12
Faculty or staff member	355	157	100	44.23	28.17	6.89
Poster with tear-offs	252	125	84	49.60	33.33	5.79
Website	399	128	74	32.08	18.55	5.10
Electronic bulletin board	258	92	57	35.66	22.09	3.93
Friend/fellow student	187	72	43	38.50	22.99	2.96
Poster with QR code	98	55	41	56.12	41.84	2.82
Banner ad	219	68	36	31.05	16.44	2.48
Table-top advertisement (eg, flyer in napkin holder or in student services office)	100	44	31	44.00	31.00	2.13
Table or booth on campus with project staff	85	38	28	44.70	32.94	1.93
Health or Wellness Fair	83	36	27	43.37	32.53	1.86
Social media (eg, Facebook, Twitter, etc)	169	48	26	28.40	15.38	1.79
Student club meeting	55	26	18	47.27	32.73	1.24
Newspaper advertisement	45	19	12	42.22	26.67	0.83
Other	76	23	12	30.26	15.79	0.83
Participating friend/family	39	14	9	35.90	23.08	0.62
Campus radio advertisement	39	9	4	23.08	10.26	0.28

Of those who did not report a most effective channel (at the *reach* level), 2961 left both survey items (channel reach and channel efficiency) blank. An additional 31 participants selected multiple channels for recruitment but did not identify one channel as most effective.

#### Channel Efficiency

The top eight channels (allowing for self-report of multiple channel exposures) included email (reported by 67.01% of the enrolled sample, 973/1452), followed distantly by courseware (LMSs; reported by 176/1452, 12.12%), faculty/staff referrals (100/1452, 6.89%), posters with tear-offs (84/1452, 5.79%), websites (74/1452, 5.10%), electronic bulletin boards (57/1452, 3.93%), friends (43/1452, 2.96%), and QR codes (41/1452, 2.82%).

A similar but different profile is observed when assessing only the self-reported *most influential* recruitment channel. In this case, the top eight channels (1292/1452, accounting for 88.98% of randomized enrolled participants) again showed email to be cited most often (with 60.54% of the sample reporting this to be the most influential, 879/1452), and again this was followed (with a substantial drop off in percentage of enrolled participant endorsement) by courseware/LMS (123/1452, 8.47%), websites (67/1452, 4.61%), faculty/staff referrals (53/1452, 3.65%), posters with tear-offs (54/1452, 3.72%), friends/family (43/1452, 2.96%), electronic bulletin boards (39/1452, 2.69%), and friends/fellow students (34/1452, 2.34%).

## Discussion

Findings from the qualitative phase of the study provided an examination of attitudes, perceived barriers, and perceived facilitators to the recruitment of community college students into an online randomized controlled trial. Common themes indicated support for the proposed electronic and online strategies, and little enthusiasm for traditional hard-copy materials such as flyers and posters. Findings support the supposition that research study recruitment at community colleges could benefit from maximizing available eHealth technology strategies [38] and strategies that include direct mass emails and links on websites and LMSs. By using these existing resources and infrastructure, community colleges could further increase recruitment opportunities within the context of community college students’ ability to privately and conveniently access interactive information from their own homes or mobile devices [24].

**Table 2:** Channel efficiency (most influential channel reported).

	Initially Reached (n=10,914)	Completed Consent (n=2696)	Enrolled and Randomized (n=1452)	% Reached Who Then Consented	Channel Efficiency (%)	% of Total Enrollees
Email	5531	1721	879	31.11	15.89	60.54
Courseware message (Blackboard, Angel, Genesis, etc)	570	237	123	41.57	21.58	8.47
Website	333	118	67	35.43	20.12	4.61
Faculty or staff member	177	83	53	46.89	29.94	3.65
Poster with tear-offs	146	77	54	52.74	36.99	3.72
Participating friend/family	214	80	43	37.38	20.09	2.96
Electronic bulletin board	173	60	39	34.68	22.54	2.69
Friend/fellow student	157	59	34	37.57	21.66	2.34
Other	253	76	34	30.04	13.44	2.34
Table or booth on campus with project staff	54	31	25	57.41	46.30	1.72
Social media (eg, Facebook, Twitter, etc)	139	39	21	28.06	15.11	1.45
Poster with QR code	36	23	18	63.89	50.00	1.24
Unreported	2954	29	18	0.98	0.61	1.24
Health or Wellness Fair	73	23	17	31.51	23.29	1.17
Student club meeting	19	12	10	63.16	52.63	0.69
Table-top advertisement (eg, flyer in napkin holder or in student services office)	28	16	9	57.14	32.14	0.62
Campus newspaper advertisement	28	9	5	32.14	17.86	0.34
Campus radio advertisement	10	3	3	30.00	30.00	0.21

Findings from the quantitative phase of the study were consistent with these themes. Results indicated that one primary channel in particular was responsible for recruiting approximately two-thirds of the final enrolled sample of 1,452 subjects: mass emails. A number of secondary channels were also influential, including the online strategies using colleges’ public websites and student-accessible LMS/courseware platforms. These results provide information that is useful for both ongoing process evaluation (maximizing successful channels and minimizing those with little yield) and outcome evaluation of recruitment channel success (channels that were successful for the final sample of enrolled subjects). By observing fluctuations in recruitment success through analysis of the self-reported influence of various channels, it is possible to make data-driven conclusions that can then influence the management of recruitment strategies, staff and resource allocation, and even costs [27]. As technology advances with increasing interactivity (data going to and coming from individuals) such as smartphone apps and wearable hardware (eg, glasses, watches, and biomedical attachments), so too will the potential to successfully and economically reach targeted special populations for recruitment purposes, including gathering specific individualized relevant statistics for various channels. Future research should continue to examine overall reach and specificity of reach to desired target populations in a variety of institutional contexts.

### Barriers to Recruitment

A number of factors have been identified as significant barriers to successful final enrollment, including high subject demands, staff turnover, champion turnover, lack of champion commitment, and a wide variance in resources and capabilities between college settings.

The demands on the subject (time and effort) from recruitment to intervention were atypically high, consisting of several time-consuming steps that involved a total of six online *stop points* (first page descriptions, study consent, baseline questionnaire assessment, study website registration, personal email access to credentials, and finally the study website). Dropouts were observed at each of these stages. This finding could mean that the study description and the outline of demands on the subjects were enough to dissuade certain types of otherwise eligible participants who simply didn’t want to *work that hard*. Conversely, the increasing participant demands and collection of inclusion criteria (consent form process, baseline questionnaire, and vendor-specific log-in procedures involving the retrieval of log-in credentials via a separate email) worked to effectively *weed out* participants who may not have truly met study inclusion criteria.

Another possibility, especially given some of the challenges of low SES populations, could be that some potential subjects had low *digital literacy*, which is defined as the total cognitive and technical abilities needed to use digital technologies for finding, evaluating, creating, and communicating information [39]. Such a skillset requires cognitive flexibility, as digital technologies and the literacies needed to navigate them constantly evolve [40]. Digitally literacy is key to functioning and adapting at home, at work, and when seeking health care.

A significant barrier that was identified was staff turnover (among project staff and among identified champions at community colleges). From semester to semester, the inconsistency in personnel necessitated repeated explanations of study details to new champions, and starting from the beginning in terms of enlisting these individuals for high-level cooperation and follow-up.

Relatedly, there was differential cooperation within individual community colleges. Despite local IRB approvals in many campuses, and approvals from college presidents and previous decision makers, a single midlevel administrator could (and often would) independently decide that our recommended recruitment channels would not be utilized. This problem was commonly the case for mass emails. As can be seen in our results, mass emails accounted for the majority of successfully reached (and recruited) study participants, but if a campus determined that they would not allow (or not frequently allow) a mass email to the student body, then that channel was limited or closed. This issue was problematic because this determination was often based on just one individual’s interpretation of their campus’ *policy*, despite the presence of evidence that their college had already agreed to assist in the proposed research strategies.

The reluctance or refusal to promote study recruitment via mass emails was the most significant barrier to recruitment, especially since (1) this channel produced the most number of recruits, and (2) there was such surprisingly little success in any of the other strategies. Strategies that at least showed steady (if not substantial) ongoing recruitment success in previous smoking cessation studies (eg, tear-off flyers, posters, face-to-face interactions, tabling), were relatively ineffective in the current study. This is an interesting finding, as the target population of mostly lower SES young adults is both understudied and underserved. What researchers and interventionists think of as *tried and true* strategies with other populations may result in a similar lack of success. In addition, newer high-technology approaches (eg, electronic bulletin boards, QR codes) were fully anticipated to have a steady and predictable recruitment rate, but these too did not result in substantive recruitment success.

Inconsistent infrastructures across campuses posed challenges to a standardized recruitment process. One campus might have mass email capabilities in an information technology department, while at another campus this might be housed in the office of the Dean or Vice President of Student Affairs, within a Health and Wellness Center, or even under the direct control of the office of the President. As discussed above, even with appropriate approvals *higher up*, a single person in charge of the mass email domain often decided whether or not to cooperate with recruitment requests. Staff turnover also made the sustainability of high-level cooperation challenging, especially from one semester to the next, when communication frequency dropped off.

### Facilitators

An important facilitator was the *adaptive design* planned flexibility [41] built into the 5-year study design to accommodate the need to improve recruitment strategies based on observed barriers and recruitment rates. As described earlier, this flexibility included refining study procedures such as the inclusion criteria, the number of targeted recruitment sites, and the length of the recruitment period.

As the data show, and as discussed above, the use of mass emails (which included the cooperation of key college campus decision-makers) was the greatest facilitator of successful recruitment. Although relatively low in yield (estimated anecdotally in the present study to typically reflect approximately 1% response from the entire student body receiving emails), the effect was predictable. A mass email to a large campus could yield up to 60 participants engaging with the study’s online process. Although low, this predictability allowed the investigators to maximize efforts towards encouraging this channel at every cooperating campus.

### Limitations

The Phase 1 qualitative work was conducted with only four campuses, all of which were in a single geographical area in Western New York. In Phase 2, community colleges (initially in Western New York, and eventually in multiple states) varied in their level of commitment to, and resources provided for, recruitment efforts. This issue resulted in wide variability in the types (and frequency) of channels used across campuses. Future research should examine standardized recruitment protocols with community colleges that are equally committed.

The lengthy subject demand process contributes to less generalization of study findings, but arguably may conversely contribute to the generalizability of findings in terms of (1) exclusion of *fake* study participants (eg, those only looking to earn the incentives), and (2) the *real-life* experience of registration and log-in credential retrieval in many popular Web-assisted tobacco interventions (and many other online experiences involving registration and log-in credentials). It is also noted that variances in *health literacy* or *computer literacy* may have accounted for differential success with online versus offline recruitment channels.

Finally, the inclusion criteria included a self-reported intention of quitting smoking within the next three months, to capture those who were in contemplation, preparation, and action phases of the Transtheoretical Model of Behavior change. Future studies are needed to assess recruitment and retention efforts for community college students who smoke and who are still in the precontemplation, maintenance, and relapse/recycle phases.

### Conclusion

This study explored details of the process and success of a variety of recruitment channels for study enrollment in a Web-assisted tobacco intervention study. Thoughtful planning and maximum flexibility are often needed to successfully meet projected study sample sizes. Analysis of relative channel efficiency across a wide variety of channels indicated a strong effect for digital recruitment promotion, consistent with the strengths of eHealth technology. One primary channel (mass emails) and a small number of secondary channels (including websites and LMSs) accounted for most of the recruitment success. Future and ongoing research is needed with such eHealth technology strategies, emerging strategies, and other electronic channels not typically leveraged by researchers (eg, text messaging, Instagram, Facebook, LinkedIn) to further maximize the yield from study recruitment efforts.

## Appendix

Multimedia Appendix 1 Email template.

Multimedia Appendix 2 Facebook.

Multimedia Appendix 3 Electronic bulletin board.

Multimedia Appendix 4 Flyer.

Multimedia Appendix 5 Key informant interview guide.

Multimedia Appendix 6 Focus group guide.

Multimedia Appendix 7 Recruitment domains, channels, and sample quotes.

Multimedia Appendix 8 CONSORT-EHEALTH v1-6.

## Abbreviations

FG: focus group
IRB: institutional review board
KII: key informant interview
LMS: Learning Management Systems
SES: socioeconomic status
QR: Quick Response
REDCap: Research Electronic Data Capture
WATI: Web-Assisted Tobacco Intervention

## References

[1] Montgomery SB, De Borba-Silva M, Singh P, Dos Santos H, Job JS, Brink TL (2015). Exploring demographic and substance use correlates of hookah use in a sample of southern California community college students. Calif J Health Promot.

[2] Berg CJ, An LC, Ahluwalia JS (2013). Dietary fat intake and exercise among two- and four-year college students: differences in behavior and psychosocial factors. Community Coll J.

[3] Bailey T, Jenkins D, Leinbach T (2005). What we know about community college low-income and minority student outcomes: descriptive statistics from national surveys.

[4] (2016). American Association of Community Colleges.

[5] Aud S, Wilkinson-Flicker S, Kristapovich P, Rathbun A, Wang X, Zhang J (2013). The Condition of Education 2013 (NCES 2013-037).

[6] Kena G, Hussar W, McFarland J, de Brey BC, Musu-Gillette L, Wang X, Zhang J, Rathbun A, WilkinsonFlicker S, Diliberti M, Barmer A, Bullock MF, Dunlop VE (2016). The Condition of Education 2016.

[7] Bragg D (2001). Community college access, mission, and outcomes: Considering intriguing intersections and challenges. Peabody Journal of Education.

[8] The White House (2009). Investing in Education: The American Graduation Initiative.

[9] The White House, Office of the Press Secretary (2015). FACT SHEET - White House Unveils America's College Promise Proposal: Tuition-Free Community College for Responsible Students.

[10] Lenk K, Erickson D, Nelson T, Winters K, Toomey T (2012). Alcohol policies and practices among four-year colleges in the United States: prevalence and patterns. J Stud Alcohol Drugs.

[11] Berg CJ, An LC, Thomas JL, Lust KA, Sanem JR, Swan DW, Ahluwalia JS (2011). Smoking patterns, attitudes and motives: unique characteristics among 2-year versus 4-year college students. Health Educ Res.

[12] VanKim N, Laska M, Ehlinger E, Lust K, Story M (2010). Understanding young adult physical activity, alcohol and tobacco use in community colleges and 4-year post-secondary institutions: A cross-sectional analysis of epidemiological surveillance data. BMC Public Health.

[13] Hasman L, Berryman D, McIntosh S (2013). NLM Informationist Grant - web assisted tobacco intervention for community college students. JESLIB.

[14] Sanem JR, Berg CJ, An LC, Kirch MA, Lust KA (2009). Differences in tobacco use among two-year and four-year college students in Minnesota. J Am Coll Health.

[15] Cohen A, Brawer F, Lombardi Jv (2008). The American Community College, 5th Edition.

[16] Pokhrel P, Little M, Herzog T (2014). Current methods in health behavior research among U.S. community college students: a review of the literature. Eval Health Prof.

[17] Floyd DL (2003). Student health: challenges for community colleges. Community Coll J.

[18] Prokhorov A, Yost T, Mullin-Jones M, de Moor C, Ford K, Marani S, Kilfoy B, Hein J, Hudmon KS, Emmons K (2008). “Look at your health”: outcomes associated with a computer-assisted smoking cessation counseling intervention for community college students. Addict Behav.

[19] James DC, Chen W, Sheu J (2007). Type of tobacco product used: are there differences between university and community college students?. J Drug Educ.

[20] Prokhorov A, Warneke C, de Moor C, Emmons K, Mullin Jones M, Rosenblum C, Hudmon KS, Gritz E (2003). Self-reported health status, health vulnerability, and smoking behavior in college students: implications for intervention. Nicotine Tob Res.

[21] Wall AF, BaileyShea C, McIntosh S (2012). ommunity college student alcohol use developing context-specific evidence and prevention approaches. Community Coll Rev.

[22] Civljak M, Stead LF, Hartmann-Boyce J, Sheikh A, Car J (2013). Internet-based interventions for smoking cessation. Cochrane Database Syst Rev.

[23] Creswell J (2002). Educational research: planning, conducting, and evaluating quantitative and qualitative research.

[24] McIntosh S, Wall AF, Johnson T, Done DH, Kurtzman JH, Ververs D, Ossip DJ (2016). Tobacco control at community colleges: context and opportunities. Tob Prev Cessation.

[25] Gajendra S, Ossip DJ, Panzer RJ, McIntosh S (2011). Implementing a smoke-free campus: a medical center initiative. J Community Health.

[26] Block RC, Tran B, McIntosh S (2011). Integrating the chronic care model into a novel medical student course. Health Educ J.

[27] McIntosh S, Ossip-Klein DJ, Spada J, Burton K (2000). Recruitment strategies and success in a multi-county smoking cessation study. Nicotine Tob Res.

[28] McIntosh S, Ossip-Klein DJ, Hazel-Fernandez L, Spada J, McDonald PW, Klein JD (2005). Recruitment of physician offices for an office-based adolescent smoking cessation study. Nicotine Tob Res.

[29] Prokhorov AV, Fouladi RT, de Moor C, Warneke CL, Luca M, Jones MM, Rosenblum C, Emmons KM, Suchanek Hudmon K, Yost TE, Gritz ER (2007). Computer-assisted, counselor-delivered smoking cessation counseling for community college students: intervention approach and sample characteristics. J Child Adoles Subst.

[30] Ossip-Klein DJ, McIntosh S, Utman CH, Burton K, Spada J, Guido JJ (2000). Smokers ages 50+: who gets physician advice to quit?. Prev Med.

[31] Strauss A, Corbin J (2007). Basics of Qualitative Research: Techniques and Procedures for Developing Grounded Theory, 3rd Edition).

[32] Corbin J, Strauss A (1990). Grounded theory research: procedures, canons, and evaluative criteria. Qual Sociol.

[33] Glaser BG, Strauss AL (1967). The discovery of grounded theory; strategies for qualitative research.

[34] Strauss A, Corbin J, Denzin NK, Lincoln YS (1994). Grounded theory methodology. Handbook of Qualitative Research.

[35] (2017). REDCap (Research Electronic Data Capture).

[36] Fiore M, Jaen C, Baker T (2008). Treating Tobacco Use and Dependence: 2008 Update Clinical Practice Guideline.

[37] Zbikowski S, Hapgood J, Smucker BS, McAfee T (2008). Phone and Web-based tobacco cessation treatment: real-world utilization patterns and outcomes for 11,000 tobacco users. J Med Internet Res.

[38] Powell J, Thorogood M, Coombes Y (2010). E-health promotion. Evaluating health promotion: practice and methods.

[39] American Library Association (2012). Digital Literacy Definition.

[40] Leu DJ, Kinzer C, Coiro J, Henry L, Alvermann DE, Unrau NJ, Ruddell RB (2013). New literacies: a dual-level theory of the changing nature of literacy, instruction, and assessment. Theoretical Models and Processes of Reading, 6th Edition.

[41] Chow S, Chang M (2008). Adaptive design methods in clinical trials - a review. Orphanet J Rare Dis.

