# Supplementary figures and images for: Recruitment of Community College Students Into a Web-Assisted Tobacco Intervention Study
Source: JMIR Res Protoc. 2017 May 8;6(5):e79. doi: 10.2196/resprot.6485 (PMC5440736; doi:10.2196/resprot.6485)

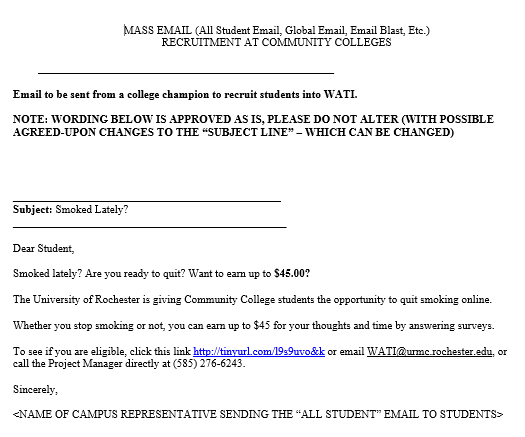

Supplement: Multimedia Appendix 1 [file resprot_v6i5e79_app1.png]

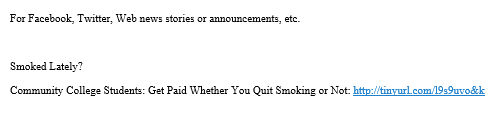

Supplement: Multimedia Appendix 2 [file resprot_v6i5e79_app2.png]

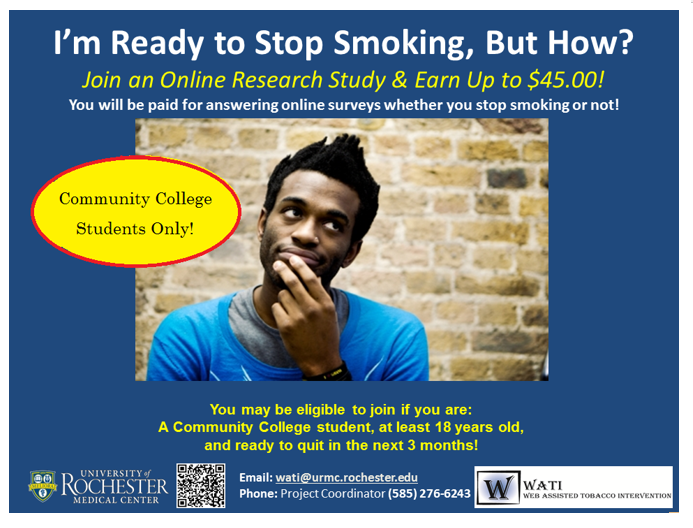

Supplement: Multimedia Appendix 3 [file resprot_v6i5e79_app3.png]

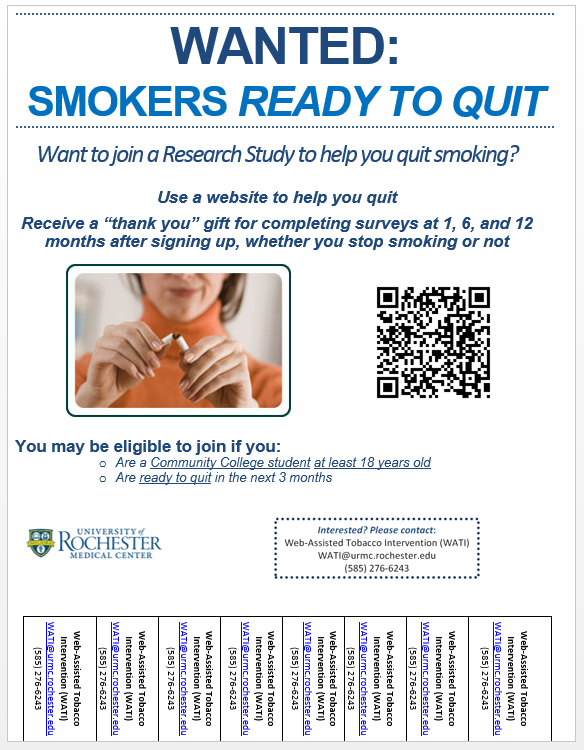

Supplement: Multimedia Appendix 4 [file resprot_v6i5e79_app4.png]
