# Supplementary material for: Recruitment of Community College Students Into a Web-Assisted Tobacco Intervention Study
Source: JMIR Res Protoc. 2017 May 8;6(5):e79. doi: 10.2196/resprot.6485 (PMC5440736; doi:10.2196/resprot.6485)
Supplement: Multimedia Appendix 5 [file resprot_v6i5e79_app5.pdf]

KII: Key Informant Interview Guides

KII Guide: Students

1. Describe smoking on this campus.

   PROMPTS: What else do you notice?

2. Describe current smoking cessation programs you are aware of.

   PROMPTS: How have you tried to quit in the past?

3. What do you know about students / peers quitting smoking?

   PROMPTS: What do you think would be successful as a campus-wide intervention? What other strategies might be successful?

4. Describe current stop smoking policies you are aware of on campus.

   PROMPTS: Any others? Restricted areas? Support for quitting?

5. Would you use a smoking cessation website? Why or why not?

   PROMPTS: Would other students use a website to help quit smoking? What makes a website interesting to you? Do you value a website's expert credibility, such as from a Health Department or doctors or other health care professionals? What features or content are most important for a health related website? What other features or content? What other technology would students use? Cell phones?

6. Recruitment for college students for research studies: Are there differences between younger and older students, and special groups like war veterans?

   PROMPTS: Are different groups of students gather or attend classes at different parts of your campus? Are different program areas located at different places? Are there student groups that are active, and would they be interested in being recruited as a group? Does time of day make a difference, for different types of groups or clubs or classes? Do some students ONLY come at night? What locations on campus would be good for posters, flyers, tent-cards, business cards, etc. (show examples)? What do you think of the "tear offs" on flyers? The QR Code? Are there distance-learning students? Do you think they would participate in a research study differently? Should they be recruited differently? Would students respond to emails to learn about a study? How? What would work best in the email, such as a link to the recruitment webpage? Specific "SUBJECT HEADER" in the email? From a health professional? From a college official? From a student or student group you are familiar with? Do you have "BlackBoard" or "Genesis" or "Angel" or other online environment for students to get campus-specific information? Would they respond to recruitment efforts on that site? How? –such as with pop-ups, or announcements, or links?

7. Would students complete web-based surveys about their smoking?

   PROMPTS: Have you used a web survey before? Which ones? [Show examples of the QR codes again, and the brief Mini-Screener from GoogleDocs] What do you think of this online survey? Too long, too short, needs more information? Will students be OK with providing their email address and phone number on an online questionnaire like this? (PRIVACY ISSUES?) What incentive would be necessary for students to complete the 3 follow-up surveys at 1, 6, and 12 months – each of which take at least 20 minutes? Is $10, $15, and $20 enough? (TOTAL = $45) What about a different way -- $10, $15, and $15 – but with a "bonus" $5 as an incentive for completing all three? Why might someone drop out completely?

8. How do you think most students would like to be reminded that it's time for a follow-up survey?

   PROMPTS: Email? Text Message? Phone call? How many times before we give up?

KII Guide: Administrators, Health/Wellness Staff, Other Key Opinion Leaders

1. Recruitment. Tell us about how you go about, or would go about recruiting students to participate in research.

   PROMPTS: What are your policies for posting posters and flyers? How have other research studies successfully conducted recruitment on campus? What other successful strategies are you aware of, or that you suggest?

2. Recruitment for college students for research studies: Tell us about different groups of students on your campus, and how you would differentially target different groups of students to participate in a research study (i.e., Older students, and special groups like war veterans)?

   PROMPTS: Do different groups of students gather or attend classes at different parts of your campus? Are different program areas located at different places? Are there student groups that are active, and would they be interested in being recruited as a group? Does time of day make a difference, for different types of groups or clubs or classes? Do some students ONLY come at night? What locations on campus would be good for posters, flyers, tent-cards, business cards, etc. (show examples)? What do you think of the "tear offs" on flyers? The QR Code? Do you have distance-learning students? Do you think they would participate in a research study differently? Should they be recruited differently?

3. Smoking as a problem on campus. Tell us about smoking on your campus and among your students? What do you observe about students groups as it relates to smoking (such as veterans, younger students, and older students)?

   PROMPTS: Where do students smoke? What else have you noticed?

4. Policies for smoking on campus (in place or in planning). What policies are in place related to smoking on your campus?

   PROMPTS: Restrictions? Cessation support? To what extent are policies related to smoking implemented and enforced? What policies are in planning stages?

5. Strategies for smoking cessation. Tell us about strategies or resources that you are aware of for your students to engage in smoking cessation?

   PROMPTS: Are there programs or classes? Do you refer smokers to health professionals? On site and off site? Do you refer smokers to the State Quitline? The website? Do you think students would use a website? Why or why not? What strategies for cessation are in planning, and/or would you like to see? Would it be possible to have referral information available, where one of the options is information about this study? Do you have "BlackBoard" or "Genesis" or "Angel" or other online environment for students to get campus-specific information? Would they respond to recruitment efforts on that site? How? - Such as with pop-ups, or announcements, or links?

6. Tell us about how we can best work with you to as we look to conduct research on your campus (if any for the study – interacting with us throughout the study).

   PROMPTS: Do you see a role for yourself? How? Are you a "champion", or are you willing to be, to support research programs and recruitment for studies? Are you aware of IRB approvals needed? Contact person? What other key contact persons should we speak to?

7. How do you see that this intervention can help your community college?

   PROMPTS: What smoking cessation features can you adopt long term for ongoing sustainable strategies? Referral strategies? Expert training?

8. Any other comments, suggestions, or questions?
