# Supplementary material for: Recruitment of Community College Students Into a Web-Assisted Tobacco Intervention Study
Source: JMIR Res Protoc. 2017 May 8;6(5):e79. doi: 10.2196/resprot.6485 (PMC5440736; doi:10.2196/resprot.6485)
Supplement: Multimedia Appendix 6 [file resprot_v6i5e79_app6.pdf]

FG: Focus Group Guide (Students)

1. Describe smoking on this campus.

   PROMPTS: What else do you notice?

2. Describe current smoking cessation programs you are aware of.

   PROMPTS: How have you tried to quit in the past?

3. What do you know about students / peers quitting smoking?

   PROMPTS: What do you think would be successful as a campus-wide intervention? What other strategies might be successful?

4. Describe current stop smoking policies you are aware of on campus.

   PROMPTS: Any others? Restricted areas? Support for quitting?

5. Would you use a smoking cessation website? Why or why not?

   PROMPTS: Would other students use a website to help quit smoking? What makes a website interesting to you? Do you value a website's expert credibility, such as from a Health Department or doctors or other health care professionals? What features or content are most important for a health related website? What other features or content? What other technology would students use? Cell phones?

6. Examine features of our recruitment process, based on interviews and resources. (Screen shots, demonstrations of web pages).

   PROMPTS: Would students respond to these strategies? Would you be interested to go to the enrollment webpage and consent process? Features available on the webpage ask students about the desirability of those features. Recruitment for college students for research studies: Are there differences between younger and older students, and special groups like war veterans? Are different groups of students gather or attend classes at different parts of your campus? Are different program areas located at different places? Are there student groups that are active, and would they be interested in being recruited as a group? Does time of day make a difference, for different types of groups or clubs or classes? Do some students ONLY come at night? What locations on campus would be good for posters, flyers, tent-cards, business cards, etc. (show examples)? What do you think of the "tear offs" on flyers? The QR Code? Are there distance-learning students? Do you think they would participate in a research study differently? Should they be recruited differently? Would students respond to emails to learn about a study? How? What would work best in the email, such as a link to the recruitment webpage? Specific "SUBJECT HEADER" in the email? From a health professional? From a college official? From a student or student group you are familiar with? Do you have "BlackBoard" or "Genesis" or "Angel" or other online environment for students to get campus-specific information? Would they respond to recruitment efforts on that site? How? –such as with pop-ups, or announcements, or links?

7. Would students complete web-based surveys about their smoking?

   PROMPTS: Have you used a web survey before? Which ones? [Show examples of the QR codes again, and the brief Mini-Screener from GoogleDocs] What do you think of this online survey? Too long, too short, needs more information? Will students be OK with providing their email address and phone number on an online questionnaire like this? (PRIVACY ISSUES?) What incentive would be necessary for students to complete the 3 follow-up surveys at 1, 6, and 12 months – each of which take at least 20 minutes? Is $10, $15, and $20 enough? (TOTAL = $45) What about a different way -- $10, $15, and $15 – but with a "bonus" $5 as an incentive for completing all three? Why might someone drop out completely?

8. How do you think most students would like to be reminded that it's time for a follow-up survey?

   PROMPTS: Email? Text Message? Phone call? How many times before we give up?
