# Supplementary material for: Recruitment of Community College Students Into a Web-Assisted Tobacco Intervention Study
Source: JMIR Res Protoc. 2017 May 8;6(5):e79. doi: 10.2196/resprot.6485 (PMC5440736; doi:10.2196/resprot.6485)
Supplement: Multimedia Appendix 7 [file resprot_v6i5e79_app7.pdf]

Multimedia Appendix 1. Recruitment domains, channels, and sample quotes.

**Domains**	**Channel**	**Sample Quote/ Source**	**Positive/Negative**
Passive			
	Posters	To be honest when I see stuff like that it seems kind of corny. I just don't even want to pay attention to it you know. (Student In Focus Group)	Negative
		[Shorten the text] because we're going from place to place we don't want to have to sit there and read something for 10 to 15 min. (Student in Focus Group)	Negative
		It would make me want to smoke if I was trying to quit and saw a cigarette and saw cigarette messages. (Student in Focus Group)	Negative
	Flyers with Tear-Offs	I was gonna say even if you have like a website like I would more likely go to a website than tear-off a piece of paper cause I'm gonna lose that paper. I would more likely look it up on my phone and like do it right there rather than carry around this piece of paper and do it later. (Student in Focus Group)	Negative
		I would only speak for myself. I would ignore something like this. (Veteran, Student Interview)	Negative
		Almost no one looks at these. (Administrator)	Negative

Electronic			
	Email	I don't know of anybody who doesn't use their student email. (Student in Focus Group)	Positive
		I personally would use my [campus email] probably… Just ease of access to me because like I'm always here usually. (Student Interview)	Positive
		With the general (community college) email, it's a must that you frequently check your email. (Student Interview)	Positive
		Our students, it's very hard to get them to use email even for official purposes … I don't know what the percentage is but they really don't use it the way we would like them to. (Administrator)	Negative
		Our students don't read their emails … You probably won't get much [if you send emails]. (Administrator)	Negative
	Courseware	I think is great. I really do. We started recently a study abroad program and that banner in Genesis that goes through was the one that caught them. (Wellness Director)	Positive
		A lot of instructors post grades and stuff on (courseware) so as soon as you go in you could put the link on the main page … so when they open it they see it right away. (Student Interview)	Positive
		People who are comfortable using (courseware) use (courseware). The instructors don't even use (courseware) and it makes it difficult to try to persuade students to when the instructors don't.	Negative

		(Student Interview)	
		What I would do is a blanket email, probably would say, our students really don't use their college email all that much. So I would email them through (courseware) because I know they read their mail there. (Educator)	Positive
	QR Codes	I'm not sure this school [would respond to QR codes] so much. I feel I don't know. I don't have a smart phone so I would take the tear-off instead. (Student Interview)	Negative
		I've never [seen one before]. (Student Interview)	Negative
		I think it's a good way to recruit people it just gonna be a good time when that becomes popular cause right now, like I said, I don't know anyone who's really doing that cause I don't even, you have to download an app right, a lot of people don't know that. (Student in Focus Group)	Negative
	Website	There would have to be something to bring them in though. It would have to be something like catchy. If it's just going to be a whole bunch of words on a page, they're gonna be like I don't have time for this. (Educator)	Negative
		I would have to first say, is this going to be a several page thing to navigate online? Am I going to go to one site and it's going to take me to another site and it's going to take me to five other sites? I'm going to have trouble signing on and registering because if that's the case, bye. (Student in Focus Group)	Negative
		You're talking about like a Facebook of just people who are trying to quit smoking? I don't think so. I just don't think the audience is big enough.	Negative

		(Student in Focus Group)	
Personal			
	Person-to-Person	You need to go where they're at, you need to go to the library, you need to go to the cafeteria, and you need to maybe even set up a table. (Student in Focus Group)	Positive
		I know a lot of people that would get into the whole… they put tables out in the forum … Probably not me personally. (Student Interview)	Negative
		Some of the faculty would be open to having you come and speak to classes … I think especially if we facilitate it. (Administrator)	Positive
